# A Rare Case of Multilevel Pott’s Disease With Coexistent Pulmonary Cavitary Tuberculosis and Neurogenic Bladder

**DOI:** 10.7759/cureus.97342

**Published:** 2025-11-20

**Authors:** Alfiia Battalova, Oksana Pron, Vikaskumar Patel, Anup Shrestha, Minhaz Murshad, Mujibur Majumder, Tutul Chowdhury

**Affiliations:** 1 Internal Medicine, Brookdale University Hospital Medical Center, Brooklyn, USA; 2 Pulmonary and Critical Care Medicine, Brookdale University Hospital Medical Center, Brooklyn, USA; 3 Hospital Medicine, Avera McKennan Hospital and University Health Center, Sioux Falls, USA

**Keywords:** low back pain, mycobacterium tuberculosis, pott’s disease, spinal mass, tuberculosis

## Abstract

Tuberculosis (TB), caused by *Mycobacterium tuberculosis,* is an infection that typically affects the lungs and is a common cause of morbidity and mortality around the world. Primary TB infection, or latent TB, is often asymptomatic; immunocompromised patients are more at risk of TB disease, which manifests with fever, fatigue, unexplained weight loss, night sweats, and productive cough. Although not common, infection can spread hematogenously to other parts of the body (e.g., kidneys and spine) and lead to extrapulmonary TB. Diagnosis made through microscopic examination for acid-fast bacilli (AFB), nucleic acid amplification test (NAAT), and mycobacterial culture is the gold standard. For extrapulmonary TB, additional invasive procedures, e.g., biopsy, are required for bacteriological confirmation. We report a case of suspected Pott's disease (spine TB) in a 21-year-old patient, who presented with back pain and lower-extremity weakness, and was found to have a spinal mass; biopsy revealed caseating granulomas. Multiple diagnostic studies failed to identify mycobacteria. Although no active TB history was reported, computed tomography (CT) of the lungs showed a cavitary lesion in the left upper lobe. The patient's condition improved after undergoing neurosurgical intervention and initiating rifampin, isoniazid, pyrazinamide, and ethambutol (RIPE) therapy.

## Introduction

Spinal tuberculosis (TB), or Pott’s disease, is the most common form of skeletal TB, yet sacral involvement remains exceptionally rare [1,2]. Diagnosis can be delayed due to its nonspecific symptoms and resemblance to malignancy or pyogenic infections. This report describes the case of a 21-year-old male with progressive neurological deficits and chronic back pain, whose imaging revealed destructive lesions in both thoracic and sacral vertebrae, which is an unusual presentation. Despite negative microbiological tests, histopathology showed caseating granulomas, and a cavitary lung lesion raised concern for disseminated TB. The patient’s recent immigration from a TB-endemic region further supported empirical anti-tubercular therapy (ATT), which led to marked clinical improvement. This case highlights the diagnostic challenges of extrapulmonary TB and the importance of early intervention based on clinical and histological findings.

## Case presentation

A 21-year-old patient presented with bilateral lower-extremity weakness for seven days, which progressed to sensory changes and fecal and urinary retention. Additionally, he had a history of upper and lower back pain for the last seven to eight months, which was progressively worsening. His social history was significant for recent immigration from Guinea to the United States, and his family history was negative; he denied a prior history of TB. The physical examination revealed a bilateral lower-extremity sensory deficit from the umbilical region downward, as well as decreased strength in the lower extremities. Strength in the upper extremities was diminished due to pain. He denied any current or previous respiratory symptoms or night sweats. Vitals in the emergency department were as follows: blood pressure (BP) 142/96 mmHg, heart rate (HR) 84 bpm, temperature 37 °C (98.6°F), respiratory rate (RR) 20 breaths per minute, and peripheral oxygen saturation (SpO_2_) 100%. Labs on admission are shown in Table 1.

**Table 1:** Labs on admission PCR: polymerase chain reaction; g/dL: grams per deciliter; /µL: per microliter; mg/dL: milligrams per deciliter; mmol/L: millimoles per liter.

Investigation	Value	Reference range
Hemoglobin (g/dL)	13.3	11.0-15.0
Hematocrit (%)	38.8	35-46
White Blood Cell (10^6^/µL)	4.5	3.8-5.3
Platelets (10^3^/µL)	277	130-400
Glucose (mg/dL)	84	80-115
Blood Urea Nitrogen (mg/dL)	10	9.8-20.1
Creatinine (mg/dL)	0.8	0.57-1.11
Sodium (mmol/L)	138	136-145
Potassium (mmol/L)	3.8	3.5-5.1
Chloride (mmol/L)	100	98-107
Bicarbonate (mmol/L)	30	23-31
Calcium (mg/dL)	9.9	8.8-10.0
Albumin (g/dL)	4.4	3.2-4.6
Magnesium (mg/dL)	2.1	1.6-2.6
COVID PCR	Negative	Negative
Prothrombin time (s)	12.2	9.8-13.4
International normalized ratio (INR)	1.1	0.85-1.15
Partial thromboplastin time (PTT, s)	32.2	24.9-35.9

**Figure 1:**
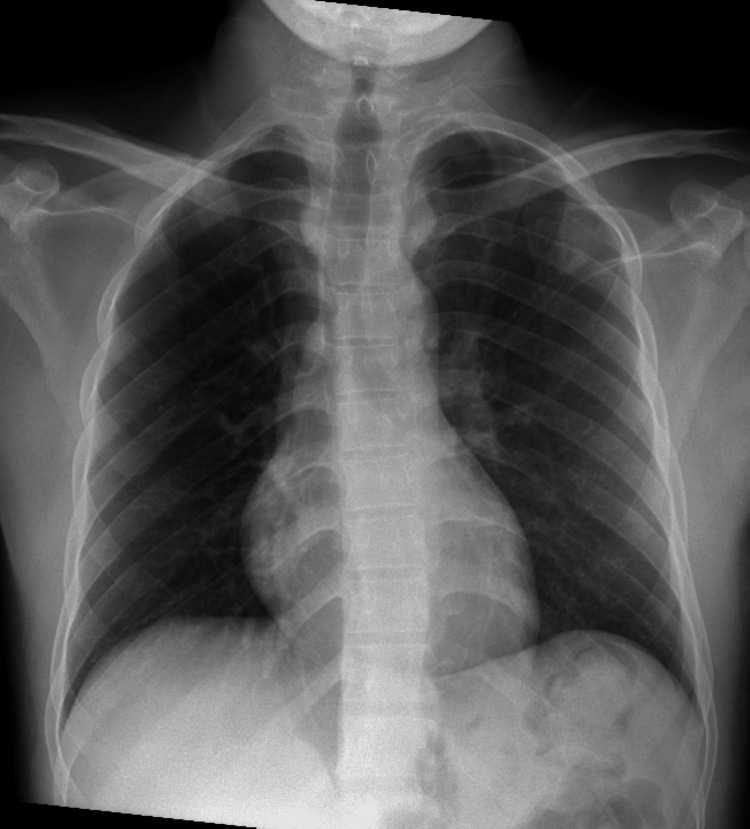
Chest X-ray on admission was normal

Chest X-ray (CXR) on day 1 was unremarkable (Figure 1). 

**Figure 2:**
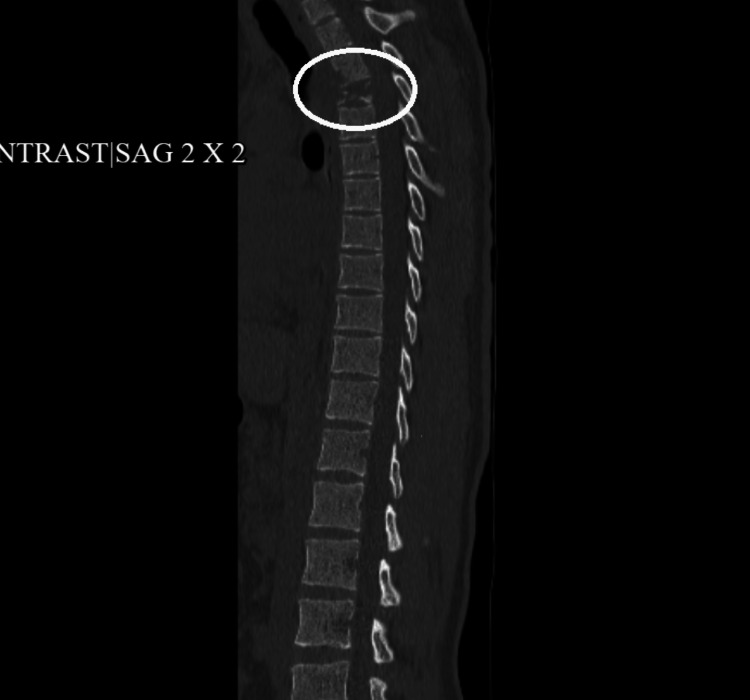
CT scan of the thoracic spine showing destruction of the T3 vertebra CT: computed tomography.

Computed tomography (CT) scan of the thoracic spine without contrast revealed destruction of most of the T3 vertebrae with a large paraspinous soft tissue mass and destruction of the inferior aspect of the T2 vertebra (Figure 2).

**Figure 3:**
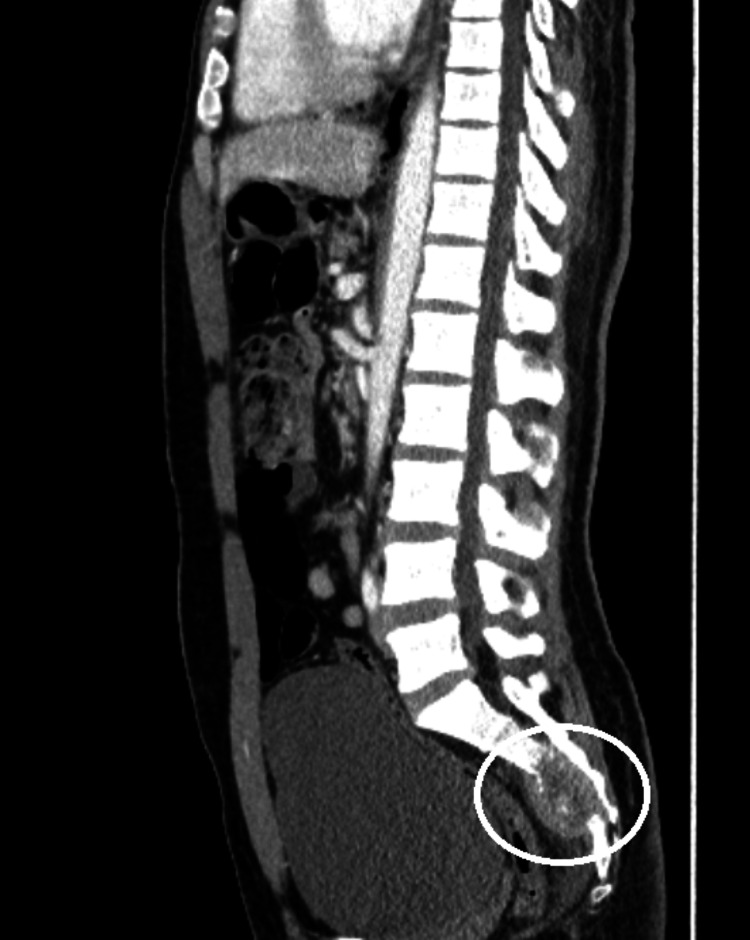
CT abdomen showing the sacrum with S3 and S4 demonstrating a lytic lesion CT: computed tomography.

CT scan of the abdomen with contrast revealed lytic lesions in the sacrum at the levels of S3 and S4 with contiguous spread involving the peri-sacral space (Figure 3); these findings were suspicious for aggressive processes such as metastatic disease. 

Neurosurgery was consulted and requested magnetic resonance imaging (MRI) of the spine.

**Figure 4:**
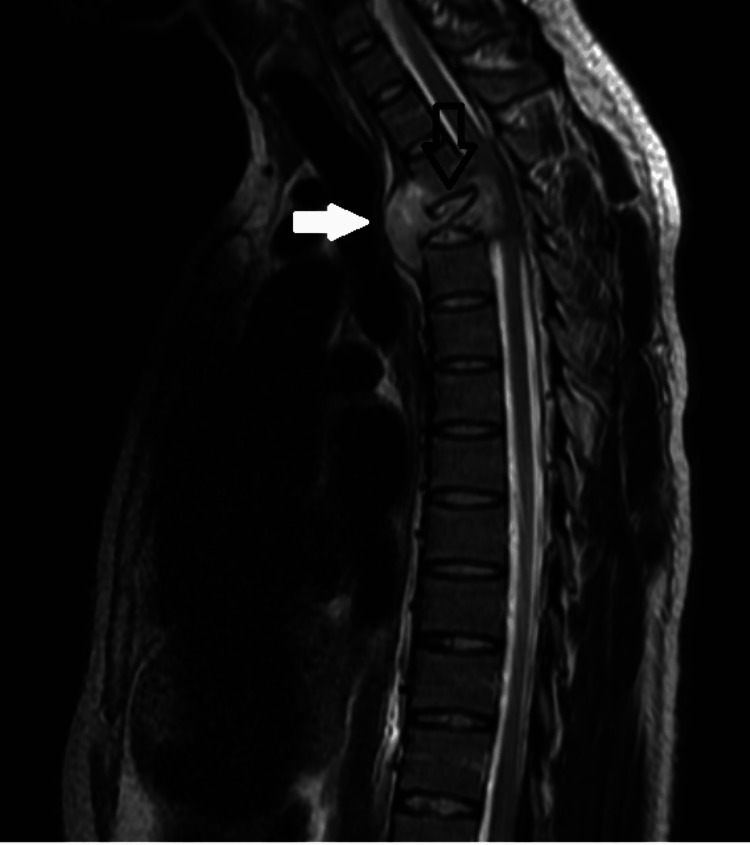
MRI showing the metastatic compression fracture (black arrow) and a prevertebral paraspinous soft tissue mass (white arrow) MRI: magnetic resonance imaging.

MRI of the thoracic region showed diffuse, low signal intensity on the T1 from the inferior half of the T2 vertebra with wedge compression of the T3 vertebra; these findings suggested metastatic compression fracture; osteomyelitis could not be excluded, but is less likely. Additionally, prevertebral paraspinous soft tissue mass and epidural mass posteriorly and anteriorly were described (Figure 4). 

**Figure 5:**
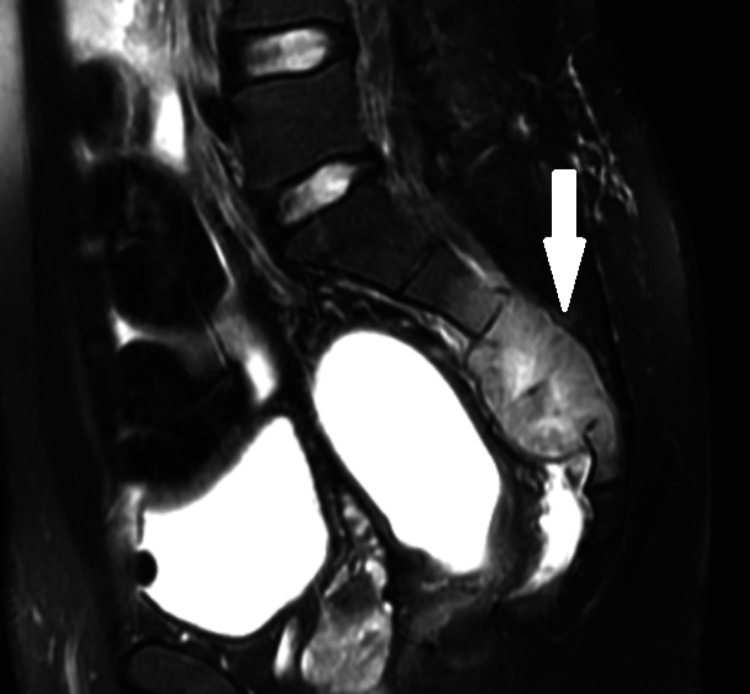
MRI of the sacrum showing S3 and S4 vertebrae with a paraspinous mass (white arrow) MRI: magnetic resonance imaging.

MRI of the sacrum showed low signal intensity from the inferior half of the S2 vertebra and most of the S3 and S4 vertebrae with a paraspinous mass primarily ventral to the spinal canal (Figure 5). These findings were most likely consistent with metastatic disease, and infection is much less likely.

**Figure 6:**
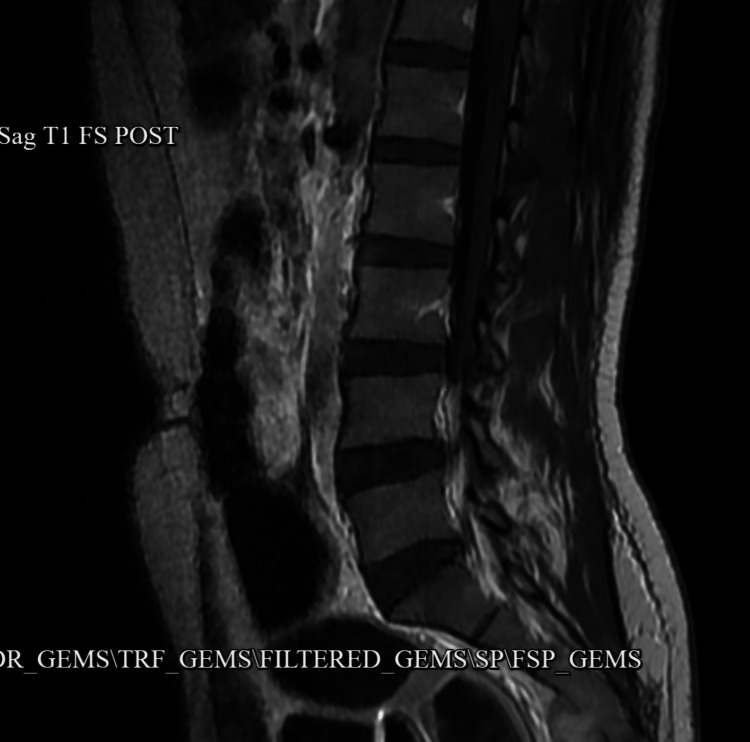
MRI of the lumbar spine was unremarkable MRI: magnetic resonance imaging.

MRI of the lumbar spine was unremarkable (Figure 6). 

**Figure 7:**
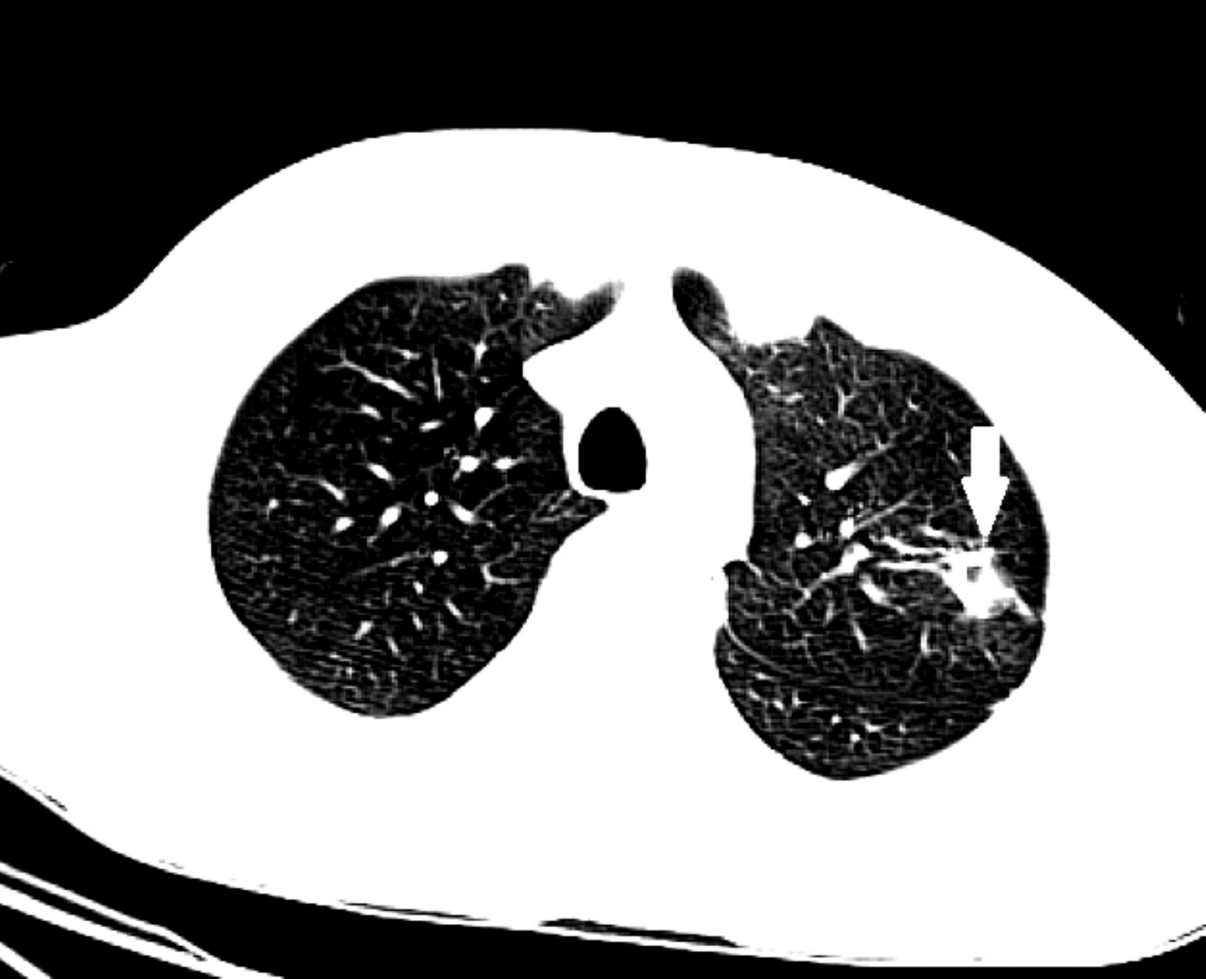
Computed tomography scan showed left upper lobe elongated nodular opacity with a cavitary component

CT scan of the chest performed on day 5 after hospitalization revealed a cavity lesion in the left upper lobe (Figure 7). 

**Figure 8:**
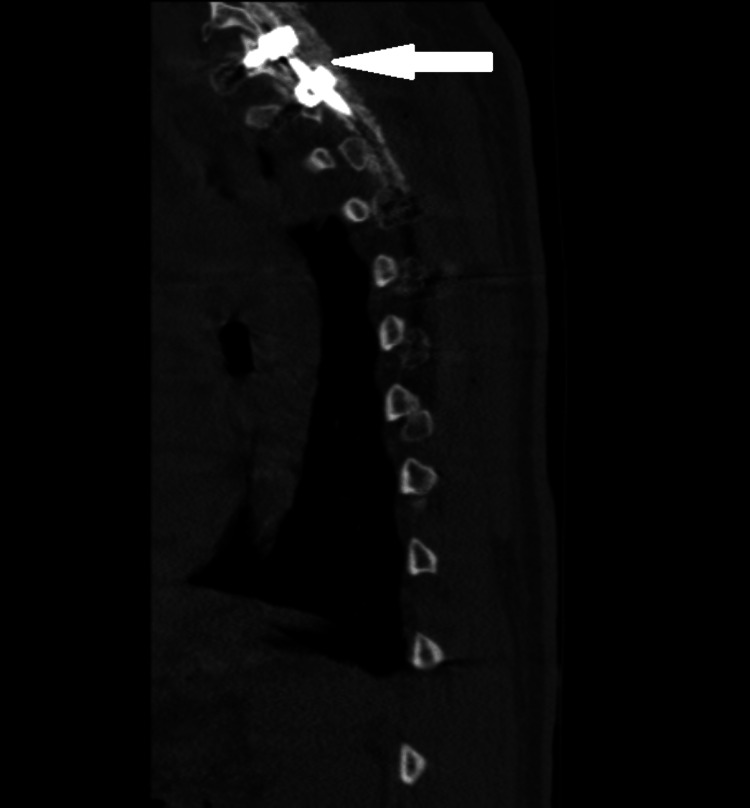
CT of the thoracic spine showing postoperative changes of T1 through T4 posterior instrumented fusion with multilevel laminectomy CT: computed tomography.

The patient was taken for spinal surgery and underwent C5-T6 posterior instrumented fusion (Figure 8)and T2-T4 laminectomies and transpedicular decompression of an epidural tumor. Procedure findings included vascular epidural tissue, which could be a tumor or phlegmon, with soft tissue without any frank pus.

Biopsies sent revealed chronic granulomatous inflammation with focal caseating-type necrosis. Acid-fast bacilli (AFB) and Grocott-Gomori methenamine silver (GMS) stains were negative for organisms. No AFB were isolated after six weeks. TB labs were sent (Table 2).

**Table 2:** TB-specific lab studies TB: tuberculosis; MTB: *Mycobacterium tuberculosis*; RIF: resistance to rifampin.

QuantiFERON TB-Gold	
NiL Gray	1.30
TB1 green	<0
TB2 yellow	0.15
Mitogen lavender	8.7
QuantiFERON-TB Gold Plus	Negative
Xpert MTB/RIF assay	MTB not detected (on 3 occasions)

MTB/RIF could not be made from the pathology or bronchoalveolar lavage samples, as it was not FDA-approved. Bronchoscopy was done, and the samples were collected from bronchoalveolar lavage. Results are shown in Table 3.

**Table 3:** Lab studies from BAL sample BAL: bronchoalveolar lavage.

Bronchial culture	Normal respiratory flora
Fungal culture	No fungus isolated at 4 weeks
Acid-fast smear	Negative
Acid-fast culture	Negative

Blood acid-fast smear and culture were negative at six weeks. Considering the presence of a cavitary lung lesion and caseating granuloma in the spine lesion, the decision was made to start empirical ATT to attempt to prevent irreversible neurological compromise. On follow-up three months after initiating ATT, the patient reported resolution of his back pain following surgical management.

## Discussion

Spinal TB (Pott’s disease) remains a significant extrapulmonary manifestation of *Mycobacterium tuberculosis*, accounting for nearly 50% of skeletal TB cases. However, simultaneous involvement of both thoracic and sacral vertebrae is exceedingly rare, with sacral TB representing less than 1% of spinal TB cases [1]. The coexistence of pulmonary cavitary lesions further complicates the clinical picture, suggesting disseminated or advanced disease. Our patient presented with thoracic and sacral vertebral destruction, myelopathy, and urinary retention - hallmarks of spinal cord compression. These neurological deficits are often late manifestations and may result from epidural abscess, vertebral collapse, or granulation tissue impinging on the spinal cord [2]. The presence of a cavitary lesion in the lung apex is consistent with active pulmonary TB, which may serve as the primary source of hematogenous spread to the spine [3].

The dual vertebral involvement raises important diagnostic considerations. Malignancy, pyogenic spondylitis, and metastatic disease must be ruled out, especially when sacral lesions are present. MRI remains the gold standard for evaluating spinal TB, offering superior soft tissue contrast and early detection of marrow edema, abscesses, and cord compression [4,5].

RIPE therapy (rifampin, isoniazid, pyrazinamide, ethambutol) remains the cornerstone of treatment, typically administered for 6-9 months depending on disease severity and response [5,6]. Surgical intervention may be warranted in cases of severe neurological compromise or spinal instability [7,8], though our patient responded favorably to medical management.

This case underscores the importance of maintaining a high index of suspicion for TB in endemic regions, even when presentations deviate from classical patterns. Multilevel spinal involvement with concurrent pulmonary disease should prompt early imaging and microbiological confirmation to prevent irreversible neurological damage. To the best of our knowledge, this is one of the few reported cases of thoracic and sacral Pott’s disease with coexistent pulmonary cavitation and neurogenic bladder, highlighting the protean nature of tuberculosis and the need for multidisciplinary management.

## Conclusions

This case illustrates the diagnostic complexity and clinical urgency of spinal TB, particularly when presenting with multilevel vertebral involvement and atypical features such as sacral lesions and neurogenic bladder. Despite negative microbiological studies, the presence of caseating granulomas and a cavitary pulmonary lesion supported a diagnosis of disseminated TB. The patient’s rapid neurological decline and imaging findings necessitated early surgical intervention and empirical ATT, which led to significant clinical improvement. Given the rarity of simultaneous thoracic and sacral involvement, this case underscores the importance of maintaining a high index of suspicion for TB in patients from endemic regions, even in the absence of classical respiratory symptoms or microbiological confirmation. It also highlights the critical role of histopathology and advanced imaging in guiding diagnosis and treatment. Early recognition and multidisciplinary management remain essential to prevent irreversible neurological damage and optimize outcomes in extrapulmonary TB.

## References

[1] Jain AK (2010). Tuberculosis of the spine: A fresh look at an old disease. J Bone Joint Surg Br.

[2] Sharma SK, Mohan A (2004). Extrapulmonary tuberculosis. Indian J Med Res.

[3] Moorthy S, Prabhu NK (2002). Spectrum of MR imaging findings in spinal tuberculosis. AJR Am J Roentgenol.

[4] World Health Organization (2010). Treatment of Tuberculosis: Guidelines for National Programmes. https://www.who.int/news/item/07-05-2010-treatment-of-tuberculosis-guidelines-for-national-programme.

[5] Jain AK, Dhammi IK (2007). Tuberculosis of the spine: A review. Clin Orthop Relat Res.

[6] Jain A, Kandwal P, Sarkar B, Mittal S, Singh V, Verma V, Maheshwari V (2023). Utility of clinicoradiological, microbiological, histopathological, and molecular methods in the diagnosis of spinal tuberculosis. Eur Spine J.

[7] Li Z, Wang J, Xiu X, Shi Z, Zhang Q, Chen D (2023). Evaluation of different diagnostic methods for spinal tuberculosis infection. BMC Infect Dis.

[8] Karthek V, Bhilare P, Hadgaonkar S, Kothari A, Shyam A, Sancheti P, Aiyer SN (2021). Gene Xpert/MTB RIF assay for spinal tuberculosis - Sensitivity, specificity and clinical utility. J Clin Orthop Trauma.

